# In Vitro and In Vivo Evaluation of a New Experimental Polydimethylsiloxane-Based Endodontic Sealer

**DOI:** 10.3390/jfb16110402

**Published:** 2025-10-28

**Authors:** Fabiola Cardoso Maldonado, Cesar Gaitan Fonseca, Carlos Bermudez Jimenez, Luis Alejandro Aguilera Galaviz, Margarita L. Martinez-Fierro, Lorena Troncoso Vazquez, Martha Eugenia Reyes Ortiz

**Affiliations:** 1 Master of Biomedical Sciences Program, Academic Unit of Odontology, Autonomous University of Zacatecas, Zacatecas 98000, Mexico; 29104759@uaz.edu.mx; 2 Faculty of Dentistry, Academic Unit of Odontology, Autonomous University of Zacatecas, Zacatecas 98000, Mexico; 3 Molecular Medicine Laboratory, Academic Unit of Human Medicine and Health Sciences, Autonomous University of Zacatecas, Zacatecas 98000, Mexico; margaritamf@uaz.edu.mx (M.L.M.-F.);; 4 General Hospital of Zone No. 1, Mexican Institute of Social Security (IMSS)*,* Zacatecas 98000, Mexico; lorena.troncoso@imss.gob.mx

**Keywords:** endodontic sealer, cytotoxicity, biocompatibility, subcutaneous implantation

## Abstract

Successful root canal treatment depends on adequate obturation with biocompatible and non-cytotoxic materials. This study evaluated the in vitro and in vivo biological characteristics of an experimental polydimethylsiloxane (PDMS)-based endodontic sealer and compared it with Silco^®^ and Sealapex^®^ cement. Human dermal fibroblasts (HDFa) were exposed to polydimethylsiloxane-based sealer eluates, Silco^®^ and Sealapex^®^, at concentrations of 1:200, 1:100, 1:50, 1:1, and undiluted eluate (1×) for 24, 48, and 72 h, and they were subcutaneously implanted in Wistar rats for 15, 30, and 45 days. Cell viability exceeded 90% at 24–48 h and remained at 85% at the highest concentration after 72 h. Sealapex^®^ showed approximately 85% viability at 24 h, over 70% at 48 h, and remained below the cytotoxicity threshold at 72 h. Silco^®^ showed a marked reduction, with values approaching 50% at 24 h. At 48 and 73 h, Silco^®^ showed a significant reduction in cell viability. Histological analysis revealed only mild acute and chronic inflammation, with no statistically significant differences over time. These results indicate that the experimental sealant demonstrates favorable biological properties suitable for further clinical evaluation.

## 1. Introduction

Endodontic treatment aims to preserve teeth affected by pulpal or periapical disease; however, treatment failure is frequently associated with persistent microbial leakage and inadequate sealing of the root canal system. Studies have reported that microleakage and reinfection remain among the leading causes of endodontic failure, highlighting the critical role of obturation materials in long-term clinical success [1,2,3,4].

One of the main objectives of endodontic therapy is the complete and accurate obturation of the root canal system. Following diagnosis, canal instrumentation and shaping must be complemented with an inert, biocompatible and dimensionally stable filling material, typically consisting of gutta-percha points in combination with an endodontic sealer [4,5]. The appropriate selection of the filling material plays a critical role in the long-term success of endodontic treatment [3]. Overall success is multifactorial, relying on thorough mechanical preparation and chemical cleaning through irrigation, as well as on adequate obturation and proper coronal restoration to maintain the integrity of the treated tooth [1,6]. Therefore, understanding the properties and performance of endodontic sealers is essential for appropriate clinical application. According to Grossman, an ideal sealer must meet physical, chemical, and biological requirements [5,7,8,9].

Endodontic sealers are essential to ensure proper obturation after cleaning and shaping of the root canal. Their primary functions include filling irregularities and micro spaces within the canal, eliminating residual bacteria, and preventing reinfection. In addition, by providing germicidal activity, they contribute to canal disinfection. Effective sealing is therefore crucial to prevent bacterial leakage and tissue debris infiltration, ensuring the long-term success of root canal treatment [4,8]. Obturation is defined as the hermetic, three-dimensional and stable sealing of the root canal system, including the apical foramen, using inert and biocompatible materials that adhere to dentin [10].

According to the Glossary of Endodontic Terms (10th edition), an endodontic sealer is a radiopaque dental material used in conjunction with a solid or semi-solid material (usually gutta-percha) to fill and seal the canal space during obturation [11]. Because gutta-percha alone cannot adapt to all anatomical complexities, endodontic sealers are responsible for filling voids and irregularities inaccessible to gutta-percha cones, creating a stable sealer-dentin-gutta-percha interface [3,12].

A wide variety of endodontic sealers are commercially available, each offering specific advantages such as antimicrobial properties, biocompatibility, radiopacity, flow, and setting time. Endodontic sealers are generally classified by chemical composition, including zinc oxide–eugenol-based, resin-based, glass ionomer-based, silicone-based, calcium hydroxide-based, bioceramic-based, and bioactive formulations [13]. However, no single material fully meets the criteria of Grossman´s “ideal sealer” [3].

Any medical device that is implanted in a living organism must satisfy structural biocompatibility, which refers to the medical interactions between an implanted medical device and the surrounding tissues [14]. Since endodontic sealers remain in intimate and prolonged contact with perirradicular tissues, biocompatibility and low cytotoxicity are essential to prevent adverse tissue responses [4,15]. Nevertheless, many biomedical materials lack the functional properties required for stable interaction with biological systems [16].

Silicone-based sealers have demonstrated favorable biological behavior, attributed to their hydrophobic methyl groups [16], low solubility, dimensional stability, expansion capacity and reduced film thickness, all of which support effective adaptation to canal walls [3,17,18,19,20].

Polydimethylsiloxane (PDMS) is a silicone-based polymer with elastic behavior; it has shown biocompatibility, elasticity and rheological versatility. Its viscoelastic nature allows it to behave as a viscous fluid at higher temperatures and as an elastic solid at lower temperatures [21]. PDMS is also inert, non-toxic, optically transparent and resistant to dimensional changes and water absorption, making it suitable for diverse biomedical applications such as maxillofacial prosthetics, synthetic fibers, coatings, adhesives and optical materials [14,16,17,21,22].

The endodontic sealer presented in this article is a new PDMS-based sealer, whose chemical formulation has been modified to evaluate the physical, chemical and biological characteristics under the rigor of international standards. The development of this endodontic sealer addresses critical requirements for successful root canal treatment. Unlike existing products, this experimental material combines radiopacity agents for clear radiographic evaluation of obturation, an antimicrobial component that inhibits the growth of oral microorganisms in contact with tissue fluids, and a chemical precursor that ensures stable bonding within the canal. These physicochemical features are designed to provide optimal sealing, reducing the risk of microleakage and minimizing reinfection risk while maintaining biological safety. Our hypothesis is that this endodontic sealer can prevent or significantly reduce microleakage in root canal.

The main objective of this article was to evaluate in vitro and in vivo biological behavior of eluates from the PDMS-based endodontic sealer in human dermal fibroblast cell culture (HDFa) at different concentrations and incubations times, as well as its biocompatibility in Wistar rat subcutaneous tissue.

## 2. Materials and Methods

### 2.1. In Vitro Study

#### 2.1.1. Cell Line 

To analyze the cytotoxicity of the endodontic sealer, the dermal fibroblast cell line HDFa (ATCC^®^ PCS-201-012; ATCC, Manassas, VA, USA) was used and cultured with the distributor's specifications y guidelines; ATCC^®^ brand fibroblast basal medium (PCS-201-030; ATCC, Manassas, VA, USA), ATCC^®^ brand fibroblast growth kit (PCS-201-040; ATCC, Manassas, VA, USA) and ATCC^®^ brand Penicillin-Streptomycin-Anfotericin B solution (PCS-999-002; ATCC, Manassas, VA, USA) under standard culture conditions: 37 °C, 100% humidity, 95% O_2_ and 5% CO_2_. In vitro culture assays were performed using HDFa human fibroblasts cultured in a 75 cm^2^ sterile culture flask (43072U; Corning Inc., Corning, NY, USA).

#### 2.1.2. Composition of Endodontic Sealers

The experimental PDMS-based endodontic sealer was synthesized at the Biomaterials Research Laboratory, Autonomous University of San Luis Potosí, San Luis Potosí, Mexico. Its formulation is based on a cross-linked polydimethylsiloxane (PDMS) matrix containing inert silica fillers and a platinum-based catalyst system. The detailed chemical composition cannot be disclosed due to ongoing patent application procedures. However, the formulation follows the requirements of ISO 6876:2012 for endodontic sealers in terms of consistency, flow, and setting characteristics [23]. For comparison, two commercially available sealers were used: Sealapex^®^ (Kerr Corporation, Orange, CA, USA), a calcium hydroxide–based sealer, and Silco^®^ (Mexican manufacturer, Xochitepec, Morelos, Mexico) a zinc oxide–eugenol–based sealer. The general compositions of all materials are summarized in Table S1 (Supplementary Material).

#### 2.1.3. Endodontic Sealer Extracts 

The materials tested were PDMS-based endodontic sealer (experimental material), Silco^®^ and Sealapex^®^, both used as comparative sealers. The protocol of extraction was followed according to ISO 10993-5:2009 [24] and 10993-12:2021 [25]. These are international standards that monitor the biological evaluation of medical devices, including chemical characterization, cytotoxicity, irritation, sensitization, systemic toxicity, genotoxicity, leachability, and local effects after implantation, among other characteristics [15].

Silco^®^ and Sealapex^®^ endodontic sealers were prepared under aseptic conditions according to the manufacturers’ instructions. For both, standardized discs (10 mm diameter × 5 mm height) were obtained by placing the freshly mixed pastes into sterile polyethylene molds and allowing them to set.

For the experimental PDMS-based endodontic sealer, the base paste and catalyst were weighed at a 1:1 ratio on an analytical balance in 2 mL Eppendorf^®^ tubes (Eppendorf AG, Hamburg, Germany). The tubes were taken to the laminar flow chamber, and the parts were mixed under sterile conditions with a sterile spatula on a glass slab, following the instructions provided by the scientific team responsible for the physicochemical synthesis of the material. The mixture was transferred into sterile molds of the same dimensions to form the discs, covered with parafilm and were incubated under 5% CO_2_ at 37 °C for 24 h to ensure complete setting.

After setting, all discs were removed from the molds and sterilized under ultraviolet (UV) light for 30 min. To obtain sealer extracts, each disc was immersed in 1 mL of fibroblast basal medium (PCS-201-030; ATCC, Manassas, VA, USA) in 2 mL Eppendorf^®^ tubes (Eppendorf AG, Hamburg, Germany) and incubated in a humidified atmosphere with 5% CO_2_ at 37 °C for 72 h. The eluates were then centrifuged for 40 s, collected using sterile syringes, and filtered through 0.22 μm sterile filters. The extracts were further sterilized under UV light for 15 min before use. Serial dilutions were prepared in culture medium to obtain final concentrations of 1:200, 1:100, 1:50, 1:1, and undiluted (1×) for the cytotoxicity assays.

#### 2.1.4. In Vitro Cytotoxicity Assay

After being cultured and upon reaching 90–100% confluence, cells were subcultured in 96-well flat-bottom plates (3797; Corning Inc., Corning, NY, USA) with 2 × 10^4^; cells per well and incubated for 24 h to allow attachment. Subsequently, the extracts were added at the respective dilutions (1:200, 1:100, 1:50, 1:1 and undiluted [1×]), incubated and the period of exposure to the extracts was quantified. At 24, 48 and 72 h after addition of the extracts, 10 μL of Alamar Blue Cell Viability Reagent (Thermo Fisher Scientific^®^, Waltham, MA, USA) were added with incubation for 2 h and the optical density was measured at wavelengths of 590 nm in a spectrophotometer Smartreader 96 (Accuris Instruments, Edison, NJ, USA). The absorbance was converted to percent cell viability with the formula provided by the manufacturer: Viability (%) = (Abs_sample/Abs_control) × 100. The basal cultured HDFa fibroblasts without endodontic sealer extract was the control group. The experiments were performed in triplicate.

### 2.2. In Vivo Study

#### 2.2.1. Murine Model

For the in vivo phase, nine healthy male Wistar rats (250–300 g), with standardized weight and age, were obtained from the Biotherium “Claude Bernard” Campus Siglo XXI, Autonomous University of Zacatecas, and maintained under controlled laboratory conditions. The characteristics of the animals followed the specifications of ISO 10993 [24,25]. The experimental design was as follows: three rats were evaluated at 15 days, three at 30 days, and three at 45 days after subcutaneous implantation. Each rat received two tubes with the experimental PDMS endodontic sealer, two with Sealapex^®^, two with Silco^®^, and one negative control tube. It should be noted that the in vivo sample size (*n* = 9) is limited, which may reduce the statistical power of the study; this is acknowledged as a limitation of the experimental design. However, in accordance with the 3Rs principle and the exploratory nature of ISO 10993 screening, this number of animals (three per time point) was considered appropriate, with multiple implants per animal to reduce inter-animal variability and minimize animal use [24,25].

#### 2.2.2. Sealer Preparation 

The endodontic sealers were weighed according to the manufacturer's instructions; in the case of the experimental sealer, it was weighed in an analytical microbalance and transferred into sterile 2 mL Eppendorf^®^ tubes (Eppendorf AG, Hamburg, Germany). The tubes were sterilized under UV light in laminar flow chamber for 15 min on each side. Each sealer was then mixed under aseptic conditions on a sterile glass slab with sterile spatula, following the manufacturer's instructions. With sterile healing forceps and spatula sterile polyethylene tubes (internal diameter: 1.0 mm; external diameter: 1.6 mm; length: 10.0 mm) were filled with the experimental PDMS endodontic sealer (base-to-catalyst ratio 1:1), Silco^®^ endodontic sealer comparator (according to manufacturer’s directions), Sealapex^®^ sealer reference (according to manufacturer’s directions), or left empty to serve as negative controls according to ISO 10993 [24,25]. The tubes were kept in an aseptic area until the time of implantation.

#### 2.2.3. Subcutaneous Implant

For the surgical procedure, and after standardizing it, the animals were weighed (Torrey® L-EQ 5/10 scale, Monterrey, Mexico) and anesthetized by inhalation of isoflurane 5–4% (lot G48E21B expiration date 30 April 2026) in a sealed chamber for approximately 2–3 min. After induction, the dorsum of each rat was shaved against the grain and aseptically prepared.

Four subcutaneous pockets were created sequentially, one in each dorsal quadrant. For each pocket, a ≈ 1 mm incision was made with a No. 3 scalpel handle and a No. 10 blade, the tissue was bluntly dissected, the polyethylene tube was inserted, and the incision was immediately closed with 4–0 nylon sutures before proceeding to the next site. The implants were distributed as follows: two tubes with the experimental PDMS endodontic sealer in the upper left quadrant, two tubes with Silco^®^ in the upper right quadrant, two tubes with Sealapex^®^ in the lower left quadrant, and one empty sealed air-filled tube (negative control) in the lower right quadrant. 

Following surgery, animals were monitored until full recovery from anesthesia and then housed in controlled conditions (22 ± 2 °C; 12 h light/dark cycle) with ad libitum food and water intake. Euthanasia was performed in groups of three rats at 15, 30 days and 45 days, respectively, following institutional and national ethical guidelines and standards. The implanted tubes with surrounding tissue were dissected and fixed in 10% buffered formalin for histological analysis. Biological waste was disposed of in compliance the corresponding norms, using designated containers for pathological residues and refrigeration until collection.

#### 2.2.4. Histological Evaluation 

Histological processing was carried out at the Interdisciplinary Research Laboratory, National School of Higher Studies (ENES), Leon Unit, National Autonomous University of Mexico (UNAM), Mexico. Biological samples were preserved in 10% buffered formalin, trimmed to obtain the most representative area of each group, and processed in a Histokinette (Leica). The tissues were embedded in paraffin and sectioned at 3–5 µm with Leica microtome. Sections were collected from a 30 °C water bath (Leica) onto glass slides and fixed on a heated plate. They were subsequently stained with hematoxylin and eosin (H&E) and mounted with epoxy resin and coverslips.

Histological evaluation was performed using the scoring system shown in Table 1 [26,27]. Each parameter was scored semi-quantitatively according to severity. Observations were conducted by a blinded expert pathologist under a Leica DM2000 optical microscope (Leica Microsystems, Wetzlar, Germany) under 10× magnification, and representative images were captured using Olympus® cellSens Standard software, version 3.2 (Olympus Corporation, Tokyo, Japan).

#### 2.2.5. Statistical Analysis

For in vitro cytotoxicity assays, data were analyzed using SigmaPlot version 12.0 (Systat Software, San Jose, CA, USA) on Windows 11. The factor analyzed was the extract dilution (1:200, 1:100, 1:50, 1:1 and 1×) within each sealer and time point (24, 48 and 72 h). Data from triplicate experiments were first tested for normality (Shapiro–Wilk) and homogeneity of variance (Levene’s test). When assumptions were met, one-way ANOVA followed by Holm–Sidak post hoc testing was applied. When assumptions were not met, the Kruskal–Wallis test followed by Dunn´s post hoc test was used. Results are expressed as mean ± standard deviation (SD) for parametric data or median (interquartile range, IQR) for non-parametric data. Statistical significance was set at *p* < 0.05. For clarity, parametric data are presented as bar graphs (mean ± SD) and non-parametric data as boxplots (median and IQR).

For in vivo biocompatibility assays, statistical analyses were performed using GraphPad Prism version 8.0 (GraphPad Software, San Diego, CA, USA) on Windows 11. Descriptive statistics were performed for each histological parameter. Qualitative data were analyzed one-way ANOVA followed by Dunnett’s multiple comparisons test to compare each experimental sealer with the negative control. Additionally, 2 × 2 contingency tables were constructed using scores of 2–4 for each histological variable, and chi-square tests were applied to compare each endodontic sealer with the control at each evaluation period.

## 3. Results

### 3.1. Cytotoxicity Test

The cell viability of fibroblasts exposed to sealer extracts at 24, 48, and 72 h is summarized in Table 2, while full statistical outputs, including normality tests, omnibus statistics and post hoc pairwise comparisons, are provided in Table S2 (Supplementary material). According to ISO 10993-5, materials are considered non-cytotoxic when cell viability remains above 70% [24]. Within this framework, the experimental PDMS-based sealer consistently preserved viability well above the cytotoxic threshold across all dilutions and times, while Silco^®^ showed values below the threshold at higher concentrations. Sealapex^®^ displayed intermediate responses. 

**Figure 1:**
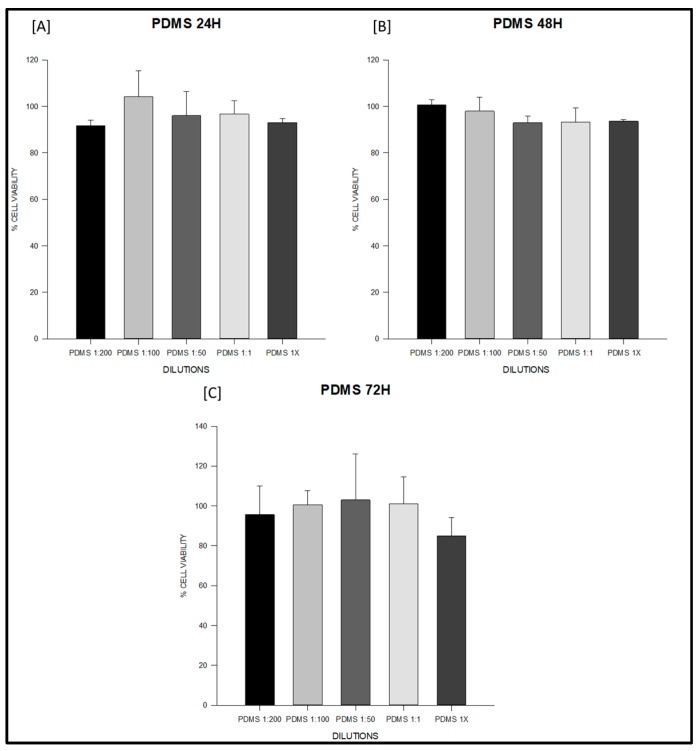
Cell viability (%) of fibroblasts exposed to the experimental PDMS-based endodontic sealer at 24, 48, and 72 h. Serial dilutions (1:200, 1:100, 1:50, 1:1, and undiluted extract, 1×) were tested. (**A**) PDMS at 24 h: all dilutions (1:200, 1:100, 1:50, 1:1, and undiluted 1×) maintained cell viability above 95%, with no significant differences between concentrations. (**B**) PDMS at 48 h: viability remained above 95% across all dilutions, indicating sustained non-cytotoxic behavior. (**C**) PDMS at 72 h: viability values ranged from 95–105%, exceeding the ISO 10993-5 threshold (>70%) at all concentrations. Data represent mean ± SD from triplicates (*n* = 3). One-way ANOVA followed by Holm–Sidak post hoc was applied when assumptions were met; otherwise Kruskal–Wallis test with Dunn’s post hoc was used. Cell viability remained above 85% at all time points and dilutions, exceeding the ISO 10993-5 threshold for non-cytotoxicity (>70%). No significant intra-material differences were detected.

**Figure 2:**
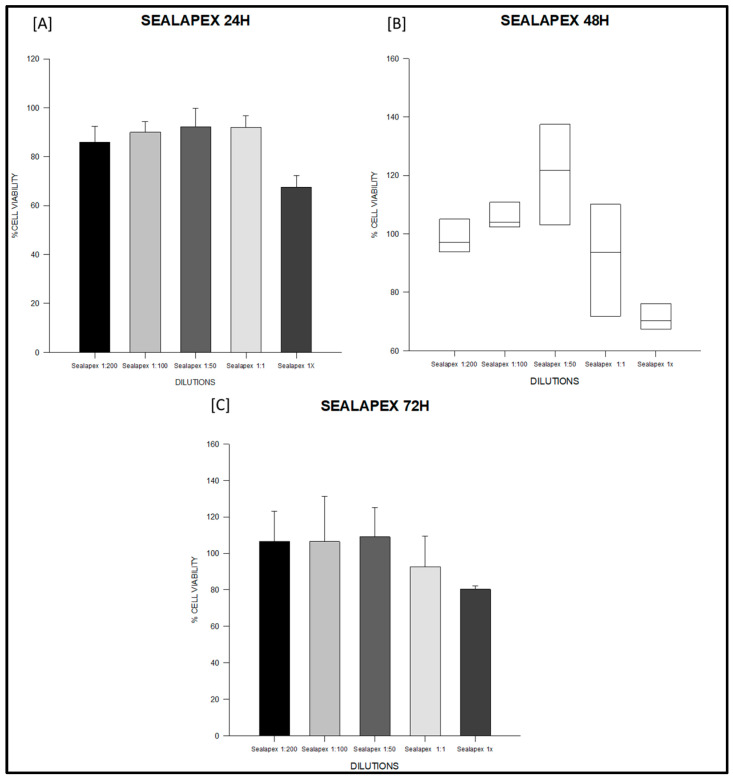
Cell viability (%) of fibroblasts exposed to Sealapex^®^ sealer at 24, 48 and 72 h. Serial dilutions (1:200, 1:100, 1:50, 1:1, and 1×) were tested. (**A**) Sealapex^®^ at 24 h: all dilutions (1:200, 1:100, 1:50, 1:1) maintained cell viability above the ISO 10993-5 threshold, while undiluted extract (1×) significantly reduced viability compared with 1:200 and 1:50. (**B**) Sealapex^®^ at 48 h: data are represented as boxplots (median, IQR, 10–90th percentile) due to non-parametric distribution; viability values remained above 70% across all dilutions. (**C**) Sealapex^®^ at 72 h: viability exceeded 90% at most dilutions, with a non-significant decline in undiluted extract (1×). Data represent mean ± SD from triplicates (*n* = 3). One-way ANOVA followed by Holm–Sidak post hoc was used when assumptions were met; otherwise, Kruskal–Wallis with Dunn’s post hoc was applied. Full statistics are provided in Table S2.

**Figure 3:**
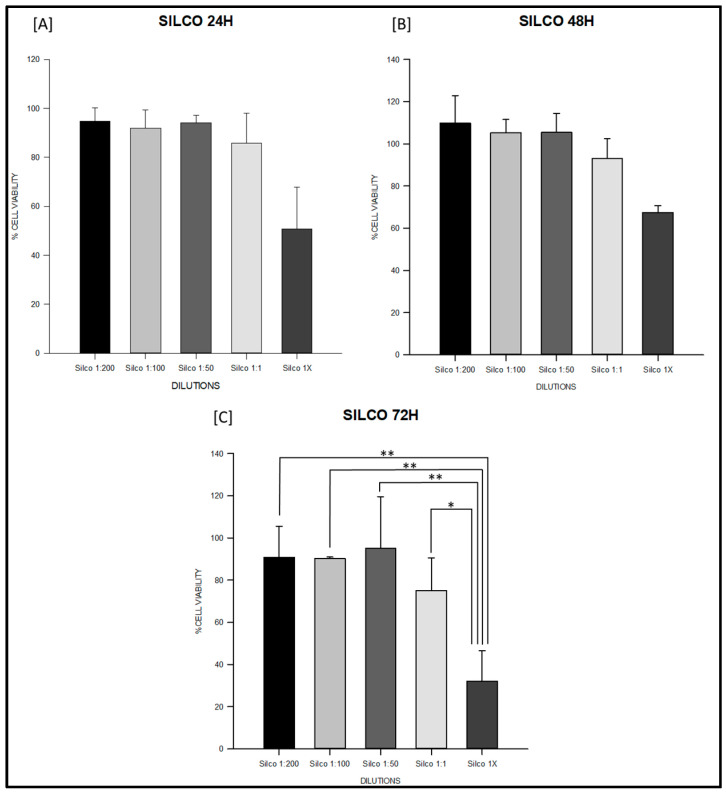
Cell viability (%) of fibroblasts exposed to Silco^®^ sealer at 24, 48 and 72 h. Serial dilutions (1:200, 1:100, 1:50, 1:1, and 1×) were tested. (**A**) Silco^®^ at 24 h: undiluted extract (1×) significantly reduced viability to approximately 50%, falling below the ISO 10993-5 cytotoxicity threshold. (**B**) Silco^®^ at 48 h: significant decreases were observed at 1:1 and 1× compared with 1:200 and 1:100, indicating a dose-dependent cytotoxic effect. (**C**) Silco^®^ at 72 h: undiluted extract reduced viability below 40%, significantly lower than all diluted conditions (* *p* < 0.05, ** *p* < 0.01), demonstrating the strongest concentration-dependent cytotoxic effect at longer exposure times. Data are shown as mean ± SD or boxplots depending on normality. Full statistical analysis is detailed in Table S2.

At 24 h, the PDMS-based sealer maintained values close to 100% across all dilutions, with no significant intra-material differences (ANOVA F = 1.27, degrees of freedom (df) = 4,10, *p* = 0.34; Table S2; Figure 1A). Sealapex^®^ extracts also remained above 85% in most dilutions; however, undiluted extract (1×) significantly decreased viability compared with 1:200 and 1:50 (F = 9.86, df = 4,10, *p* = 0.002; Table S2; Figure 2A). Silco^®^ presented a marked reduction at 1×, with viability dropping to approximately 50%, significantly lower than diluted conditions (F = 9.75, df = 4,10, *p* = 0.002; Table S2) and below the ISO 10993 threshold (*p* < 0.01; Figure 3A).

At 48 h, PDMS continued to show stable values above 95% with no significant differences among dilutions (F = 2.11, df = 4,10, *p* = 0.16; Table S2; Figure 1B). Sealapex^®^ exhibited variable viability, ranging from ~85% to >130%, but all values remained above the 70% threshold; statistical testing did not detect significant intra-material differences at this time point (Kruskal–Wallis H = 7.23, df = 4,10, *p* = 0.19; Table S2; Figure 2B). Silco^®^, however, demonstrated significantly reduced viability at 1:1 and 1× compared with its diluted extracts (F = 11.44, df = 4,10, *p* = 0.001; Table S2; Figure 3B), with 1× remaining below the cytotoxicity cut-off.

At 72 h, PDMS preserved viability between 95–105% across dilutions, all above the ISO threshold (F = 0.75, df = 4,10, *p* = 0.57; Table S2; Figure 1C). Sealapex^®^ extracts generally remained above 90%, although a decline was noted for undiluted extract without reaching statistical significance (F = 1.57, df = 4,10, *p* = 0.25; Table S2; Figure 2C). Silco^®^ exhibited the most pronounced cytotoxic effect: viability dropped below 40% at 1×, significantly lower than at 1:200, 1:100, and 1:50 (F = 8.09, df = 4,10, *p* = 0.004; Table S2; Figure 3C). Taken together, these results demonstrate that the PDMS-based sealer was consistently non-cytotoxic under ISO 10993 criteria, maintaining viability above 70% across time points and dilutions. Sealapex^®^ showed an intermediate, mostly non-cytotoxic profile with occasional reductions in undiluted extracts. In contrast, Silco^®^ exerted significant and concentration-dependent cytotoxicity, with viability values frequently below the ISO threshold, particularly at high concentrations and longer exposures.

In vitro cytotoxicity results are summarized in Table 2 and illustrated for each material in Figure 1, Figure 2 and Figure 3. These figures highlight the different response patterns: consistently high viability for the PDMS-based sealer, intermediate values for Sealapex^®^, and a concentration-dependent reduction for Silco^®^. Together, these data confirm that only Silco^®^ reached cytotoxic levels (viability <70%) according to ISO 10993, while the PDMS-based sealer remained non-cytotoxic across all dilutions and time points.

### 3.2. Biocompatibility Test

Based on the histological characteristics evaluated (Table 1), a frequency table was constructed (Table 3) summarizing the observations made by the pathologist. Descriptive statistics, one-way analysis of variance (ANOVA), and Dunnett’s multiple comparison test were performed, with no statistically significant differences observed (*p* > 0.05). In addition, 2 × 2 contingency tables were constructed using scores of 2, 3 and 4 for each parameter in order to apply the chi-square (χ^2^) test to compare each sealer with the negative control at each evaluation period. No statistically significant differences were found. According to ISO 10993, these findings indicate that all materials tested elicited an acceptable tissue response comparable to the negative control [25].

**Figure 4:**
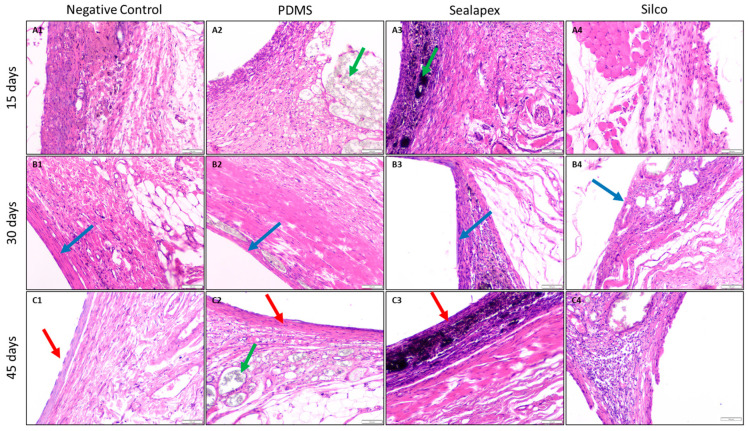
Photomicrographs of hematoxylin and eosin (H&E)-stained sections showing the capsule at the tissue–material interface at 15, 30 and 45 days. Images correspond to the negative control, PDMS-based experimental sealer, Sealapex^®^, and Silco^®^. ×10 magnification. The green arrow indicates material, the blue arrow indicates capsule, and the red arrow indicates the capsule epithelialization zone. At 15 days, capsule borders were irregular and dominated by inflammatory infiltrates (mostly histiocytes), with little to no fibrosis. At 30 days, the capsule appeared more fibrous and compact, with greater fiber organization and reduced inflammation. By 45 days, epithelialization was evident with stratified squamous epithelium and minimal inflammatory infiltrate.

Figure 4 presents the total number of histological observations recorded during the study. For the PDMS-based experimental endodontic sealer, inflammatory responses at 15 and 30 days were characterized by acute and chronic patterns, comparable to the control group. At 45 days, 33.3% of the samples exhibited chronic inflammation, while 66.6% presented both acute and chronic inflammation.

Regarding the severity of inflammation, based on the number of inflammatory cells, 66.6% of the samples at 15 days showed a low count (<25 cells) and 33.3% a mild count (25–50 cells). At 30 and 45 days, all samples demonstrated a moderate count (>50–75 cells), findings consistent with the control group.

The extent of inflammation revealed that, at 15 days, two rats presented inflammatory cells confined to the superficial capsule layer, while in one rat they were limited to the fibrous capsule. At 30 days, all samples displayed inflammatory cells in the superficial capsule layer. At 45 days, two rats again showed superficial capsule involvement, and one rat exhibited cells limited to the fibrous capsule. Overall, these findings closely resembled the control group.

Evaluation of the connective tissue capsule demonstrated that, at 15 and 30 days, an immature fibrous capsule was observed, as in the control group. At 45 days, one rat presented a thin capsule (<150 µm), while the remaining two showed an immature capsule, consistent with the control. Concerning collagen fiber morphology, 33.3% of the samples at 15 days showed normal fibers, while 66.6% exhibited mild irregularity. At 30 days, all samples presented normal collagen morphology. At 45 days, one sample displayed normal morphology, one showed mild irregularity, and one moderate irregularity. These findings indicate that fibrous tissue formation secondary to contact with the PDMS-based endodontic sealer was not significant.

A foreign body reaction, assessed through the presence of giant cells, was absent in all samples and time points, results identical to the control group.

Capillary reactions included congested vessels, perivascular material, and vascular proliferation. Congested vessels were slightly present at 15 and 30 days, and at 45 days they were mild in one rat and moderate in two rats. Material in the perivascular zone was observed in one rat at 15 days and in two rats at 45 days, likely due to displacement by normal animal movements. Vascular proliferation, defined as >25 vascular structures per field, was observed in all samples at 45 days, similar to the control group.

Finally, no calcification, coagulative necrosis, or macrophage infiltration was detected in any group at any evaluation time.

## 4. Discussion

The success of root canal treatment depends on efficient instrumentation, thorough cleaning, proper obturation, and the final restoration of the tooth. In the obturation phase, endodontic sealers play a key role by facilitating the placement of gutta-percha points, filling root canal irregularities, preventing leakage through sealing, and providing other essential functions [2,3].

Among the characteristics of an ideal endodontic sealer are physical, chemical, and biological properties. It is crucial that an endodontic sealer fulfills most of the requirements described by Grossman [13]. In particular, the biological properties demand that the material be non-cytotoxic, biocompatible, and well tolerated by periradicular tissues [4,15]. To evaluate these biological responses, cytotoxicity and subcutaneous implant tests are recommended according to the international standard ISO-10993 [4,24].

The objective of this study was to evaluate the biological profile of a novel PDMS-based endodontic sealer in a preclinical phase, in a fibroblast cell culture and using a Wistar rat subcutaneous implantation model. Because obturation materials may come into direct contact with periradicular tissues following apical extrusion, their cytotoxicity and biocompatibility must be carefully assessed [4]. Therefore, it is necessary that the endodontic sealer be biocompatible and tolerated by the periradicular tissues [1,28,29].

According to ISO 10993, cell viability below 70% at the highest extract concentration is indicative of cytotoxic effects [24,30]. In this study, the experimental PDMS-based endodontic sealer consistently maintained fibroblast viability above the 70% threshold across all dilutions and time points, confirming its non-cytotoxic profile. Statistical analysis (ANOVA or Kruskal–Wallis with appropriate post hoc testing) revealed no significant differences among dilutions of the PDMS sealer, underscoring its stability in vitro. Sealapex^®^ exhibited intermediate behavior, with occasional reductions in undiluted extracts but overall maintaining viability above 70%. By contrast, Silco^®^ (zinc oxide–eugenol) displayed a clear dose- and time-dependent cytotoxicity, with undiluted extracts reducing viability to ~40–50%, significantly below the ISO cut-off. These findings highlight the favorable biological performance of PDMS-based formulations compared with conventional sealers.

The favorable profile of PDMS can be explained by its chemical inertness and structural stability. Cross-linked polydimethylsiloxane matrices are hydrophobic and release minimal soluble components, which limit cytotoxic leachables. These mechanisms are consistent with earlier investigations. Collado-Gonzalez et al. reported that periodontal ligament stem cells (hPDLSCs) exposed to eluates of GuttaFlow Bioseal^®^, GuttaFlow2^®^, MTA Fillapex^®^, and AH Plus^®^ exhibited high levels of proliferation, spreading, and attachment when cultured on GuttaFlow Bioseal^®^ discs. Silicone-based endodontic sealers such as GuttaFlow2^®^ and GuttaFlow Bioseal^®^ were less cytotoxic than MTA Fillapex^®^ and AH Plus® [31]. Moreover, endodontic sealer extracts showed time- and dose-dependent effects on hPDLSCs. Another study comparing freshly mixed and set sealers found that GuttaFlow® exhibited the lowest cytotoxicity and even stimulated human periodontal ligament fibroblast proliferation [32].

Accardo et al. further confirmed the favorable biological profile of silicone-based endodontic sealers. Their study compared the in vitro cytotoxicity of GuttaFlow2^®^, original GuttaFlow^®^, and fast-setting GuttaFlow^®^ with the epoxy resin-based AH Plus Jet^®^. Cell viability of periodontal ligament cells was significantly reduced by AH Plus Jet^®^ at 1, 2, and 4 weeks, whereas all GuttaFlow^®^ variants showed high cell survival rates at all time intervals [33]. These findings are in agreement with our results.

Conversely, the cytotoxicity of zinc oxide–eugenol systems like Silco^®^ is well documented and attributed to eugenol release, which has membrane-disruptive and mitochondrial inhibitory effects, as well as potential to induce oxidative stress and apoptosis [3]. This explains the dose-dependent cytotoxicity observed in our assays, where Silco^®^ produced the lowest viability values and consistently exceeded the cytotoxicity threshold set by ISO 10993 [24,25].

The present findings are also consistent with those reported by Sequeira et al. [34], who evaluated PDMS-based endodontic sealers (GuttaFlow^®^ and GuttaFlow Bioseal^®^) and observed cell viabilities consistently above 80% in fibroblast cultures after 24 and 48 h, classifying them as non-cytotoxic according to ISO 10993-5 [34]. In that study, the authors attributed the excellent biological performance of PDMS-based materials to their chemical inertness, minimal leachability, and stable polymer network, which reduce the release of residual monomers and by-products. These results parallel those obtained in the present work, where the experimental PDMS-based sealer also maintained fibroblast viability above 95% across all dilutions and exposure times, confirming the characteristic biocompatibility of silicone-based endodontic materials.

The in vivo implantation assay corroborated the in vitro findings. The PDMS-based sealer elicited only mild inflammatory responses, comparable to the control group, throughout the 45-day evaluation. No necrosis, calcification, or foreign-body reaction was observed, and fibrous capsule maturation followed a normal progression. These tissue-level findings agree with the high cell viability observed in vitro and reinforce the overall biological safety of the material.

Because coronal leakage is another critical factor that can compromise endodontic treatment [35], it is important for sealers to exhibit favorable physicochemical properties. The PDMS-based endodontic sealer evaluated here showed adequate hydrophobicity and flow, supporting its classification as a suitable endodontic sealer. Considering both its high cell viability in vitro and its favorable tissue response in vivo, this material demonstrates adequate biological properties to be considered a promising endodontic sealer.

## 5. Conclusions

The experimental PDMS-based endodontic sealer demonstrated consistent non-cytotoxicity in fibroblast cultures, with viability values remaining above the ISO 10993 threshold across all dilutions and time points [24,25]. In vivo, the material elicited only mild and resolving tissue responses in a rat subcutaneous model, comparable to the negative control and without evidence of necrosis, calcification, or foreign-body reaction. Together, these results provide robust preclinical evidence of the biocompatibility of the PDMS-based formulation.

In contrast to the dose-dependent cytotoxicity observed with the zinc oxide–eugenol sealer, the PDMS-based material proved stable and biologically well tolerated. These findings position PDMS-based sealers as a safer alternative to conventional formulations, supporting their further development and potential clinical use.

Beyond confirming its favorable biological profile, this study expands the limited body of evidence on PDMS-based sealers by combining in vitro and in vivo testing within the same investigation. While these results are encouraging, clinical trials remain essential to validate the long-term safety and effectiveness of PDMS-based sealers in human applications.

## 6. Limitations and Perspectives

This study presents certain limitations that should be acknowledged. The cytotoxicity assessment was performed in fibroblast cell cultures, and the in vivo evaluation was limited to a subcutaneous rat model, which does not fully reproduce the complex periapical environment present in humans. Although the PDMS-based endodontic sealer demonstrated low cytotoxicity and favorable tissue compatibility, these results should be interpreted with caution when extrapolated to clinical conditions.

Future investigations should focus on more comprehensive evaluations. First, it is important to assess potential genotoxic effects of the material to ensure genomic stability in surrounding tissues. Likewise, cell proliferation and differentiation assays, particularly in human periodontal ligament stem cells and osteoblast-like cells, would provide further insight into the biological interactions of the endodontic sealer. Additional studies should also evaluate the material’s influence on mineralization potential and periapical tissue regeneration.

Finally, well-designed clinical trials in patients are essential to validate these preclinical findings and determine the sealer’s long-term performance, safety, and effectiveness under clinical conditions. Incorporating both basic and translational research will be key to establishing the PDMS-based endodontic sealer as a reliable alternative in endodontic therapy.

## Figures and Tables

**Table 1:** Scoring criteria for histological evaluation of tissue response to endodontic sealers. Summarizes the histological analysis evaluation criteria. Six key features were assessed: inflammation, fibroblastic reaction, fibrous tissue formation, capillary reaction, calcification and coagulative necrosis. Each parameter was categorized and scored on a scale from 0 to 4, where 0 indicates absence or normal tissue response, and higher scores reflect increasing severity or intensity of the observed reaction.

Feature	Category	Score				
		0	1	2	3	4
Inflammation	I. Type	Absence of acute and chronic	Chronic	Acute	Presence of both	
	II. Severity	No detected inflammatory cells	Cell count < 25 (sparse)	Cell count 25 –50 (mild)	Cell count > 50–75 (moderate)	Cell count > 75 (severe)
	III. Extent	No detected inflammatory cells	Inflammatory cells just at the superficial layer of the capsule	Cells limited to the fibrous capsule	Cells beyond the fibrous capsule	No capsulation/cells are limited to interface
Fibroblastic reaction	Connective tissue capsule	Absent- without capsule	Immature form Dispersed fiber	Thin capsule (<150 µm)	Thick capsule (≥150 µm)	
Fibrous tissue formation	Thickness of connective tissue capsule	Normal collagen fiber morphology	Mild collagen fiber irregularity	Moderate collage fiber irregularity	Severe collagen fiber irregularity	
Capillary reaction	Congested blood vessels	Absent	Mild	Moderate	Severe	
	Perivascular material	Absent	Present			
	Vascular proliferation	No significant vascular proliferation	Number of vascular structures in field > 25	Number of vascular structures in field 25–50	Number of vascular structures in field > 50	
Calcification		Absent	Present			
Coagulative necrosis		Absent	Present			

**Table 2:** Cell viability (%) after exposure to endodontic sealer extracts at 24, 48 and 72 h. Data are expressed as mean ± SD from triplicates (*n* = 3) at 24, 48, and 72 h. Dilutions tested were 1:200, 1:100, 1:50, 1:1, and undiluted extract (1×). Statistical analysis was performed per time point: if assumptions of normality and homogeneity were met, one-way ANOVA followed by Holm–Sidak post hoc was applied; otherwise, Kruskal–Wallis test with Dunn’s post hoc was used. Complete test statistics and adjusted *p*-values are provided in Table S2.

Sealer	Time	*n*	1:200 (Mean ± SD)	1:100 (Mean ± SD)	1:50 (Mean ± SD)	1:1 (Mean ± SD)	1× (Mean ± SD)
PDMS	24 h	3	91.64 ± 2.43	104.05 ± 11.28	95.94 ± 10.30	96.64 ± 5.62	92.97 ± 1.82
	48 h	3	100.52 ± 2.15	97.91 ± 6.02	92.93 ± 2.82	93.17 ± 6.18	93.57 ± 0.77
	72 h	3	95.65 ± 14.29	100.63 ± 7.12	103.00 ± 23.10	100.94 ± 13.55	84.91 ± 9.15
Sealapex	24 h	3	85.90 ± 6.54	90.01 ± 4.35	92.14 ± 7.58	92.07 ± 4.61	67.54 ± 4.79
	48 h	3	99.17 ± 7.68	106.33 ± 5.98	120.43 ± 22.99	91.25 ± 25.65	71.56 ± 5.97
	72 h	3	106.55 ± 16.66	106.47 ± 24.72	109.08 ± 16.11	92.65 ± 16.84	80.33 ± 1.70
Silco	24 h	3	94.81 ± 5.27	91.93 ± 7.32	94.05 ± 2.99	85.66 ± 12.24	50.67 ± 17.16
	48 h	3	109.88 ± 13.02	105.23 ± 6.38	105.43 ± 9.05	93.12 ± 9.29	67.45 ± 3.34
	72 h	3	90.82 ± 14.61	90.30 ± 0.67	95.15 ± 24.48	75.12 ± 15.43	32.04 ± 14.43

**Table 3:** Frequencies of histologic features observed in subcutaneous tissue samples. Shows the characteristics evaluated in the Wistar rat tissues at 15, 30 and 45 days in response to the different endodontic sealers used, as well as the control group; which consisted of the implantation of an empty polyethylene tube to obtain the evaluation of tissue response due to the traumatic effect of the implant in the subcutaneous tissue.

Sealer	PDMS, *n* (%)			SILCO®, *n* (%)			SEALAPEX®, *n* (%)			CONTROL, *n* (%)		
Score	15 Days	30 Days	45 Days	15 Days	30 Days	45 Days	15 Days	30 Days	45 Days	15 Days	30 Days	45 Days
Type of inflammation												
0	0 (0)	0 (0)	0 (0)	0 (0)	0 (0)	0 (0)	0 (0)	0 (0)	0 (0)	0 (0)	0 (0)	0 (0)
1	0 (0)	0 (0)	1 (33.3)	1 (33.3)	0 (0)	3 (100)	0 (0)	0 (0)	2 (66.6)	1 (33.3)	0 (0)	3 (100)
2	0 (0)	0 (0)	0 (0)	0 (0)	0 (0)	0 (0)	0 (0)	0 (0)	0 (0)	0 (0)	0 (0)	0 (0)
3	3 (100)	3 (100)	2 (66.6)	2 (66.6)	3 (100)	0 (0)	3 (100)	3 (100)	1 (33.3)	2 (66.6)	3 (100)	0 (0)
Severity of inflammation												
0	0 (0)	0 (0)	0 (0)	0 (0)	0 (0)	0 (0)	0 (0)	0 (0)	0 (0)	3 (100)	0 (0)	0 (0)
1	2 (66.6)	3 (100)	3 (100)	2 (66.6)	3 (100)	3 (100)	2 (66.6)	3 (100)	3 (100)	0 (0)	3 (100)	3 (100)
2	1 (33.3)	0 (0)	0 (0)	1 (33.3)	0 (0)	0 (0)	1 (33.3)	0 (0)	0 (0)	0 (0)	0 (0)	0 (0)
3	0 (0)	0 (0)	0 (0)	0 (0)	0 (0)	0 (0)	0 (0)	0 (0)	0 (0)	0 (0)	0 (0)	0 (0)
4	0 (0)	0 (0)	0 (0)	0 (0)	0 (0)	0 (0)	0 (0)	0 (0)	0 (0)	0 (0)	0 (0)	0 (0)
Extent of inflammation												
0	0 (0)	0 (0)	0 (0)	0 (0)	0 (0)	0 (0)	0 (0)	0 (0)	0 (0)	0 (0)	0 (0)	0 (0)
1	2 (66.6)	3 (100)	2 (66.6)	2 (66.6)	3 (100)	2 (66.6)	2 (66.6)	3 (100)	3 (100)	3 (100)	3 (100)	3 (100)
2	1 (33.3)	0 (0)	1 (33.3)	0 (0)	0 (0)	1 (33.3)	0 (0)	0 (0)	0 (0)	0 (0)	0 (0)	0 (0)
3	0 (0)	0 (0)	0 (0)	1 (33.3)	0 (0)	0 (0)	1 (33.3)	0 (0)	0 (0)	0 (0)	0 (0)	0 (0)
4	0 (0)	0 (0)	0 (0)	0 (0)	0 (0)	0 (0)	0 (0)	0 (0)	0 (0)	0 (0)	0 (0)	0 (0)
Connective tissue capsule												
0	0 (0)	0 (0)	0 (0)	0 (0)	2 (66.6)	0 (0)	0 (0)	0 (0)	0 (0)	0 (0)	0 (0)	0 (0)
1	3 (100)	3 (100)	2 (66.6)	2 (66.6)	1 (33.3)	2 (66.6)	2 (66.6)	3 (100)	2 (66.6)	3 (100)	3 (100)	3 (100)
2	0 (0)	0 (0)	1 (33.3)	0 (0)	0 (0)	1 (33.3)	1 (33.3)	0 (0)	1 (33.3)	0 (0)	0 (0)	0 (0)
3	0 (0)	0 (0)	0 (0)	1 (33.3)	0 (0)	0 (0)	0 (0)	0 (0)	0 (0)	0 (0)	0 (0)	0 (0)
Thickness of the connective tissue capsule												
0	1 (33.3)	3 (100)	1 (33.3)	2 (66.6)	1 (33.3)	2 (66.6)	0 (0)	3 (100)	1 (33.3)	2 (66.6)	1 (33.3)	3 (100)
1	2 (66.6)	0 (0)	1 (33.3)	0 (0)	2 (66.6)	1 (33.3)	3 (100)	0 (0)	2 (66.6)	1 (33.3)	2 (66.6)	0 (0)
2	0 (0)	0 (0)	1 (33.3)	1 (33.3)	0 (0)	0 (0)	0 (0)	0 (0)	0 (0)	0 (0)	0 (0)	0 (0)
3	0 (0)	0 (0)	0 (0)	0 (0)	0 (0)	0 (0)	0 (0)	0 (0)	0 (0)	0 (0)	0 (0)	0 (0)
Congested blood vessels												
0	0 (0)	0 (0)	0 (0)	1 (33.3)	0 (0)	1 (33.3)	0 (0)	1 (33.3)	1 (33.3)	0 (0)	3 (100)	1 (33.3)
1	3 (100)	3 (100)	1 (33.3)	1 (33.3)	3 (100)	1 (33.3)	2 (66.6)	2 (66.6)	1 (33.3)	3 (100)	0 (0)	2 (66.6)
2	0 (0)	0 (0)	2 (66.6)	1 (33.3)	0 (0)	1 (33.3)	1 (33.3)	0 (0)	0 (0)	0 (0)	0 (0)	0 (0)
3	0 (0)	0 (0)	0 (0)	0 (0)	0 (0)	0 (0)	0 (0)	0 (0)	1 (33.3)	0 (0)	0 (0)	0 (0)
Perivascular material												
0	2 (66.6)	3 (100)	1 (33.3)	1 (33.3)	3 (100)	1 (33.3)	3 (100)	2 (66.6)	2 (66.6)	3 (100)	3 (100)	3 (100)
1	1 (33.3)	0 (0)	2 (66.6)	2 (66.6)	0 (0)	2 (66.6)	0 (0)	1 (33.3)	1 (33.3)	0 (0)	0 (0)	0 (0)
Vascular proliferation												
0	3 (100)	3 (100)	0 (0)	1 (33.3)	3 (100)	1 (33.3)	3 (100)	3 (100)	1 (33.3)	3 (100)	3 (100)	1 (33.3)
1	0 (0)	0 (0)	3 (100)	2 (66.6)	0 (0)	2 (66.6)	0 (0)	0 (0)	1 (33.3)	0 (0)	0 (0)	2 (66.6)
2	0 (0)	0 (0)	0 (0)	0 (0)	0 (0)	0 (0)	0 (0)	0 (0)	0 (0)	0 (0)	0 (0)	0 (0)
3	0 (0)	0 (0)	0 (0)	0 (0)	0 (0)	0 (0)	0 (0)	0 (0)	1 (33.3)	0 (0)	0 (0)	0 (0)
Calcification												
0	3 (100)	3 (100)	3 (100)	3 (100)	3 (100)	3 (100)	3 (100)	3 (100)	3 (100)	3 (100)	3 (100)	3 (100)
1	0 (0)	0 (0)	0 (0)	0 (0)	0 (0)	0 (0)	0 (0)	0 (0)	0 (0)	0 (0)	0 (0)	0 (0)
Coagulative necrosis												
0	3 (100)	3 (100)	3 (100)	3 (100)	3 (100)	3 (100)	3 (100)	3 (100)	3 (100)	3 (100)	3 (100)	3 (100)
1	0 (0)	0 (0)	0 (0)	0 (0)	0 (0)	0 (0)	0 (0)	0 (0)	0 (0)	0 (0)	0 (0)	0 (0)

## Data Availability

The original contributions presented in this study are included in the article/Supplementary Materials. Further inquiries can be directed to the corresponding author.

## Supplementary Materials

The following supporting information can be downloaded at https://www.mdpi.com/article/10.3390/jfb16110402/s1, Table S1: Composition and manufacturer information of the endodontic sealers tested in the study. Table S2: Statistical analysis of fibroblast viability after exposure to endodontic sealer extracts.
