# Supplementary material for: In Vitro and In Vivo Evaluation of a New Experimental Polydimethylsiloxane-Based Endodontic Sealer
Source: J Funct Biomater. 2025 Oct 28;16(11):402. doi: 10.3390/jfb16110402 (PMC12653106; doi:10.3390/jfb16110402)
Supplement: Supplementary file 1 [file jfb-16-00402-s001.zip › jfb-3856394-supplementary.pdf]

Table S1. Composition and manufacturer information of the endodontic sealers tested in the study.

Sealer	Main components	Manufacturer	Type
PDMS-based experimental sealer	Polydimethylsiloxane-based (experimental formulation)	Biomaterials Research Laboratory, Autonomous University of San Luis Potosí,Mexico.	Experimental formulation
Sealapex®	Calcium hydroxide, barium sulfate, zinc oxide, salicylate resin, titanium dioxide	Kerr Corporation, Orange, CA, USA	Calcium hydroxide–based
Silco®	Zinc oxide	Mexican manufacturer, Mexico	Zinc oxide–eugenol–based

Note: The detailed composition of the experimental PDMS-based sealer cannot be disclosed due to ongoing patent protection procedures. The formulation is under evaluation for intellectual property registration and will be made available upon patent publication.

Table S2. Statistical analysis of fibroblast viability after exposure to endodontic sealer extracts. Normality was tested using Shapiro–Wilk. When assumptions were met, one-way ANOVA followed by Holm–Sidak post-hoc was applied; otherwise Kruskal–Wallis with Dunn's post-hoc was used. For each sealer and time point (24, 48, and 72 h), the table presents mean ± SD of triplicates (n = 3) for each dilution (1:200, 1:100, 1:50, 1:1, 1×), the outcome of normality testing, significant pairwise comparisons, and adjusted p-values. Differences considered significant are indicated (*p < 0.05; **p < 0.01; ***p < 0.001).

SEALER	TIME	*n*	1:200 (Mean ± SD)	1:100 (Mean ± SD)	1:50 (Mean ± SD)	1:1 (Mean ± SD)	1× (Mean ± SD)	Normality test (Shapiro-Wilk)	Singificant comparisons	Holm-Sidak method
PDMS	24 hours	3	91.64 ± 2.43	104.05 ± 11.28	95.94 ± 10.30	96.64 ± 5.62	92.97 ± 1.82	p = 0.439	ns (ANOVA F= 1.27, df= 4,10, p=0.34)	/
	48 hours	3	100.52 ± 2.15	97.91 ± 6.02	92.93 ± 2.82	93.17 ± 6.18	93.57 ± 0.77	p = 0.742	ns (ANOVA F= 2.11, df= 4,10, p=0.16)	/
	72 hours	3	95.65 ± 14.29	100.63 ± 7.12	103.00 ± 23.10	100.94 ± 13.55	84.91 ± 9.15	p = 0.354	ns (ANOVA F= 0.75, df= 4,10, p=0.57)	/
Sealapex	24 hours	3	85.90 ± 6.54	90.01 ± 4.35	92.14 ± 7.58	92.07 ± 4.61	67.54 ± 4.79	p = 0.628	Significant differences (ANOVA F= 9.86, df= 4,10, p = 0.002)	1:50 vs. 1:200 (p=0.75)<br>1:1 vs. 1:200 (p= 0.71)<br>1:10 vs. 1:200 (p= 0.87)<br>1:50 vs. 1:100 (p= 096)<br>1:1 vs. 1:100 (p= 0.89)<br>1:50 vs. 1:1 (p= 0.98)
	48 hours	3	99.17 ± 7.68	106.33 ± 5.98	120.43 ± 22.99	91.25 ± 25.65	71.56 ± 5.97	p = 0.29	ns (Kruskal–Wallis H=7.23, df=4,10 p=0.124)	/
	72 hours	3	106.55 ± 16.66	106.47 ± 24.72	109.08 ± 16.11	92.65 ± 16.84	80.33 ± 1.70	p = 0.49	ns (ANOVA F= 1.57, df= 4,10, p = 0.25)	/

Silco	24 hours	3	94.81 ± 5.27	91.93 ± 7.32	94.05 ± 2.99	85.66 ± 12.24	50.67 ± 17.16	p = 0.70	Significant differences (ANOVA F= 9.75, df= 4,10, p = 0.002)	1:200 vs. 1:1 (p=0.88) 1:50 vs. 1:1 (p=0.87) 1:100 vs. 1:1 (p=0.92) 1:200 vs. 1:100 (p= 0.98) 1:50 vs. 1:100 (p= 0.96) 1:200 vs. 1:50 (p= 0.92)
	48 hours	3	109.88 ± 13.02	105.23 ± 6.38	105.43 ± 9.05	93.12 ± 9.29	67.45 ± 3.34	p = 0.89	Significant differences (ANOVA F= 11.44, df= 4,10, p = 0.001)	1:200 vs. 1:1 (p=0.22) 1:50 vs. 1:1 (p= 0.46) 1:100 vs. 1:1 (p= 0.41) 1:200 vs. 1:100 (p= 0.89) 1:200 vs. 1:50 (p=0.79) 1:50 vs. 1:100 (p= 0.97)
	72 hours	3	90.82 ± 14.61	90.30 ± 0.67	95.15 ± 24.48	75.12 ± 15.43	32.04 ± 14.43	p = 0.79	Significant differences (ANOVA F= 8.09, df= 4,10, p = 0.004)	1:1 vs. 1x (p= 0.05) 1:50 vs. 1:1 (p= 0.63) 1:200 vs. 1:1 (p= 0.76) 1:100 vs. 1:1 (p= 0.74) 1:50 vs. 1:100 (p= 0.97) 1:50 vs. 1:200 (p= 0.93) 1:200 vs. 1:100 (p= 0.96)
